# The Accuracy of Point-of-Care Ultrasound in Detecting Small Bowel Obstruction in Emergency Department

**DOI:** 10.1155/2018/3684081

**Published:** 2018-04-04

**Authors:** A. Pourmand, U. Dimbil, A. Drake, H. Shokoohi

**Affiliations:** Department of Emergency Medicine, The George Washington University School of Medicine and Health Sciences, Washington, DC, USA

## Abstract

Radiological imaging plays an essential role in the evaluation of a patient with suspected small bowel obstruction (SBO). In a few studies, point-of-care ultrasound (POCUS) has been utilized as a primary imaging modality in patients with suspected SBO. POCUS has been shown to be an accurate tool in the diagnosis of SBO with multiple research studies noting a consistent high sensitivity with a range of 94–100% and specificity of 81–100%. Specific sonographic findings that increase the likelihood of SBO include dilatation of small bowel loops > 25 mm, altered intestinal peristalsis, increased thickness of the bowel wall, and intraperitoneal fluid accumulation. Studies also reported that emergency physicians could apply this technique with limited and short-term ultrasound training. In this article, we aim to review the sensitivity and specificity of ultrasound examinations performed by emergency physicians in patients with suspected SBO.

## 1. Introduction

Computed tomography (CT) scan, magnetic resonance imaging (MRI), and plain radiography are widely used in the ED to image patients with a high pretest probability of SBO [1–3]. As reported by Kidmas et al., the accuracy of plain abdominal radiograph in diagnosing SBO varies from 50% to 92% and is used mostly in developing countries as the initial imaging tool. They also noted that CT scan has the advantage of determining the cause and predicting the location of obstruction [4].

The current established standard of care is to perform a CT scan when suspicious for an acute small bowel obstruction. However, this is associated with increased radiation exposure, delayed time to diagnosis, and increased cost. In a recent meta-analysis of imaging modalities to diagnose SBO, Taylor and Lalani concluded that CT scans are limited by the need to find the transition point between dilated bowel loops and decompressed loops prior to imaging [5]. Furthermore, review of different cases emphasized that CT is high in cost and requires a certain expertise level from the radiologist [5]. Upon examining the use of MRI for SBO diagnosis, Taylor and Lalani found that the increased time needed to perform the scan, and the limited availability of MRI centers, made this choice impractical in an acute care setting [5]. As for X-rays, Taylor and Lalani, and commentary provided by Carpenter and Pines, agreed that plain abdominal radiography is limited in diagnosing and/or excluding SBO [5, 6].

In recent years with a wide application of point-of-care ultrasound (POCUS) in the ED, ultrasound has been utilized in the diagnosis of patients with suspected SBO in a few studies. Due to its ease of use, low cost, increased accessibility, and high accuracy reported in these studies POCUS has the potential to reduce, but not overcome, many of inherent limitations of traditional imaging. The use of POCUS for a patient with suspected SBO is compelling due to the potential to reduce the use of CT scans, which would decrease cost, limit contrast agent utilization, and result in decreased cumulative imaging time.

In this article, we will discuss the accuracy of POCUS as an optimal option to diagnose SBO at the bedside in the ED. Specifically, we will review the literature where providers have used sonography as an alternative initial imaging test to evaluate patients with a suspected SBO.

## 2. Methods

A systematic literature review using PubMed, MEDLINE, Scopus, and CINAHL databases was performed using the search terms “Small Bowel Obstruction”, “point-of-care ultrasound”, “POCUS”, “Ultrasound”, and “SBO” from January 1990 to May 2017. Only studies written in English were included. All literature identified by the search strategy was considered for inclusion based on eligibility and quality. All types of studies and designs, including case reports and case series, were considered for inclusion. The bibliographies of included studies were reviewed to identify additional references. Two authors for inclusion independently reviewed identified studies. A third author was consulted to resolve any discrepancies that arose when reviewing the literature. Abstracts, unpublished data, editorials, and duplicate articles were also excluded.

## 3. Small Bowel Obstruction Ultrasound Technique

Ultrasound examination of the small bowel usually is performed in supine position. A 2.5- to 5.0-MHz curvilinear probe or 3.5–5 MHz phased array transducer is often used for this application. A 7.0- to 12.0-MHz linear transducer, which facilitates high-resolution imaging, may be used for a thin patient or for better assessment of more superficial loops and the free fluids between bowel loops. The loops of small bowel are scanned in a general sweep from the epigastrium across the mid abdomen down to the pelvis. However, considering the generalized abdominal tenderness and the possibility of air fluid levels precluding appropriate imaging, a real-time survey may start in the transverse plane in the left upper quadrant. Gentle but adequate graded compression may apply to displace gas and bowel contents.

**Figure 1:**
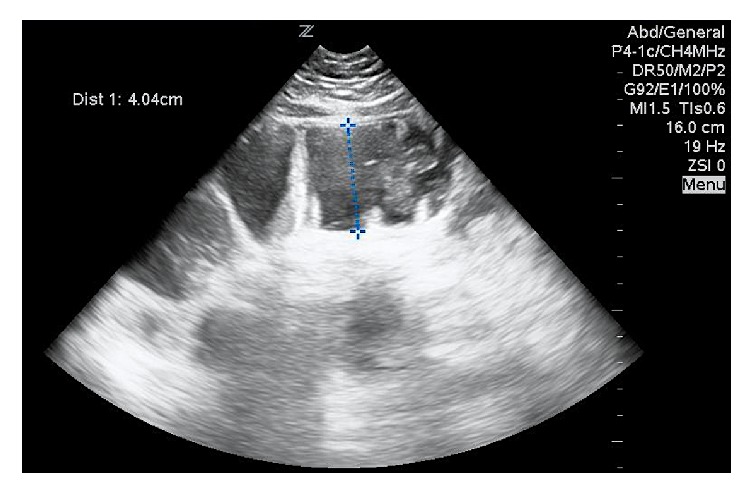
Ultrasound image using a phased array transducer shows a dilated fluid-filled loop of bowel, with a width of more than 4 cm in the left lower quadrant compatible with a small bowel obstruction.

**Figure 2:**
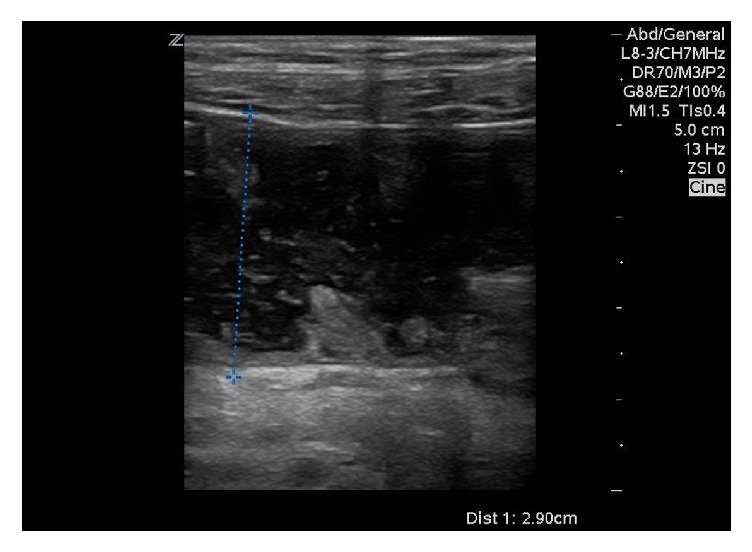
Ultrasound image using a high frequency linear transducer shows a dilated loop of bowel, with a width of 2.9 cm in the left lower quadrant compatible with a small bowel obstruction.

The specific diagnostic criterion for an ultrasound diagnosis of SBO varies in the medical literature; however most publications agree on a triple most common feature. These include (1) multiple fluid-filled dilated (>25 mm) noncompressible bowel loops juxtaposed to a collapsed bowel segment, (2) localized edema of the bowel wall with increased thickness, and (3) free fluid between the dilated loops [14] (Figures 1 and 2).

Occasionally, POCUS may be useful in determining the cause of obstruction and the subsequent severity. For example, lack of peristalsis, prominent bowel wall thickening, the presence of intraperitoneal free fluid, and a distended bowel segment on ultrasound are all indicative of a probable bowel infarction [14].

## 4. Acquiring Proficiency in POCUS

POCUS can be learned quickly and successfully performed by emergency providers after a short-term training [7]. In order to examine the accuracy of nonspecialized residents in diagnosing SBO, Ünlüer et al. reported that four third-year EM residents underwent a 3-hour didactic course and a 3-hour hands-on abdominal sonography-training program taught by a senior radiologist. The course was specifically geared toward recognizing the diagnostic imaging criteria for SBO. Following this training module, these residents spent 6 months imaging patients with suspected SBO using an ultrasound with a 3.5 MHz convex transducer. These patients underwent another ultrasound performed by blinded third-year radiology residents. Ünlüer et al. concluded that the SBO diagnoses made by emergency medicine (EM) residents with just 6 hours of training were 98% accurate and were comparable to results from radiology residents [7]. Similarly, in the study by Jang et al., EM residents, with only 10 minutes of didactic time and previous experience with only 5 SBO ultrasounds, diagnosed SBO with high levels of accuracy (Table 1) [8].

## 5. Accuracy of Ultrasound in Small Bowel Obstruction

POCUS has been shown to be an accurate tool in the diagnosis of SBO with multiple research studies noting a consistently high sensitivity and specificity in diagnosis (Table 1). While each study used slightly different standards, diagnostic approach was generally defined as the presence of the aforementioned SBO diagnostic criteria.

Barzegari et al. reported that the presence of dilated bowel (>25 mm) had the highest specificity among the other criteria in diagnosing intestinal obstruction [9] (Table 2). They showed that decreased bowel peristalsis had the highest sensitivity (100%) among the other variables, but a relatively low specificity (67.4%). Lastly, the presence of intraperitoneal fluid individually had the lowest sensitivity of all (4.5%), but a high specificity of 88.4% [9]. Several studies agreed in the accuracy US to diagnose small bowel obstruction (Table 3) [8–11].

Because of the disagreement between the sonographic criteria needed to diagnose SBO, Dickman et al. noted that while identifying dilated bowel loops is essential, there is an increased likelihood for diagnostic accuracy when there is also abnormal peristalsis. This study also reported that sonography has the potential to be used as an alternative method to identify SBO [15]. Dickman et al. recommended the use of ultrasounds at the bedside given the lack of ionizing radiation, the decreased length of stay for the patient, and the ease of use of POCUS in crowded EDs [15].

## 6. Discussion

The results of current studies suggest that because of its high specificity, POCUS is a useful modality in identifying dilated loops of bowel in patients with suspected SBO. Utilizing POCUS may reduce the number of CT scans needed to render a correct diagnosis of SBO and expedite the surgical management and care of patients in the ED. However, considering the lower sensitivity of POCUS, negative ultrasound findings should be interpreted with caution when evaluating these patients, as a negative result may not necessarily be interpreted as a negative diagnosis.

To examine the accuracy of US to diagnose SBO and cost saving and the time benefit associated with this imaging modality, Ogata et al. evaluated 50 patients with clinical and radiographic findings that were suggestive of a bowel obstruction. In this study, Ogata et al. found the sensitivity and specificity of the sonographic diagnosis of intestinal obstructions to be 88% and 96%, respectively. However, this statistical analysis included both small bowel and large bowel obstructions. For SBO specifically, ultrasound identified this diagnosis in 20 patients with only one patient having a false positive result. Lastly, this study highlighted that using sonography to identify SBO could result in earlier surgical intervention and a wider span of time in which to manage the issue without surgery. This has the potential to reduce costs and the length of hospital stay for the patient. Ogata et al. calculated the latter to currently be an average of 5 days for patients that do not require surgery and an average of 13 days for patients that do [16].

Sonography has the potential to determine the cause of small bowel ileus through specific findings [17]. In a literature review analysis examining ultrasound use in various small bowel diseases, Kralik et al. found that sonographic imaging can distinguish between the two types of ileus—mechanical and paralytic [17]. Additionally, sonographic imaging was able to both diagnose and to classify bowel obstructions. Specifically, Hollerweger et al. found that ultrasound can correctly determine the cause in a significant number of cases. The study emphasized that, in the case of neoplasm, IBD, incarcerated abdominal wall hernia, and intussusception, there is an increased likelihood of visualizing the cause of obstruction with ultrasound. In order to image the cause, the provider should look in the region of the transition between the dilated and collapsed bowel loops. Bowel wall thickening in this area hints at a neoplasm. Per this study, causes of obstruction difficult to identify on ultrasound include scarring, adhesions, anastomotic stenosis, volvulus, and ischemia [18].

## 7. Limitation

While POCUS has quite of few advantages associated with use in the diagnosis of gastrointestinal pathologies, there are certain limitations as well. First, sonography is more accurate in diagnosing complete SBO and is limited in the diagnosis of partial SBO [19]. Also, with ultrasound it is difficult to find the transition point between dilated and compressed bowel loops and to properly distinguish between potential causes of obstruction [19].

## 8. Conclusions

Point-of-care ultrasound can be used as an optimal option for the diagnosis and early management of small bowel obstruction in the ED. Studies reviewed in this article suggested that POCUS has a high specificity in detecting dilated loops of bowel, leading to the diagnosis of SBO. The findings suggest that POCUS has a comparable accuracy to CT scan in patients with suspected SBO and can be utilized as an optimal first imaging of choice at the bedside in the ED. Further research is needed to move beyond the use of US as either an adjunct or an alternative and to implement it as the sole primary imaging tool for SBO diagnosis.

## Figures and Tables

**Table 1:** Statistical analysis of using POCUS to diagnose SBO.

Study	# of Pts	Sensitivity	Specificity	PPV	NPV
Ünlüer et al. [7]	174	97.7%	92.7%	-	-
Jang et al. [8]	76	93.9%	81.4%	-	-
Barzegari et al. [9]	113	100%	78.5%	82.4%	100%
Musoke et al. [10]	70	93%	100%	100%	73%
Schmutz et al. [11]	123	95%	82.1%	-	-

**Table 2:** Using the presence of dilated bowels on US to diagnose SBO.

Study	# of Pts	Dilated loops of bowel		Interloops free fluids		Abnormal peristalsis	
		Sensitivity	Specificity	Sensitivity	Specificity	Sensitivity	Specificity
Ünlüer et al. [7]	174	94.2%	93.8%	x	x	x	x
Jang et al. [8]	76	90.9%	83.7%	x	x	27.3	97.7%,
Barzegari et al. [9]	113	97.7%	100%	4.5%	88.4%	100%	67.4%

**Table 3:** Sonographic evaluation of SBO.

Author	Design	*N*	Findings	Conclusion
Ünlüer et al. [7]	Prospective US versus CT and XR	174	No significant difference between EM and radiology residents in diagnosing BO using US.	With proper training of EM residents, their diagnostic accuracy of BO using US can be comparable to those done by radiology residents.

Jang et al. [8]	Prospective US versus CT and XR	76	US showed that the presence of dilated loop of bowel had a sensitivity and specificity of 90.9% and 83.7%, respectively, and the presence of absent peristalsis had a sensitivity and specificity of 27.3% and 97.7%, respectively.	US showed superiority over plain radiographs in detecting SBO.

Musoke et al. [10]	Prospective	70	US showed a sensitivity of 93%, specificity of 100%, PPV of 100%, and NPV of 73%.	Not only does US show promises in diagnosis, but it may play a role in detecting patients who need emergent intervention such as those with strangulation.

Ko et al. [12]	Retrospective	54	US is better than plain radiographs in diagnosing SBO and in detecting the level and cause of obstruction.	US can be helpful in diagnosing SBO when other modalities are not readily available.

Grassi et al. [13]	Retrospective	150	US not only detects the obstruction, but it can detect if this obstruction is caused by a functional or obstructive cause, and it can detect the level of severity.	Using US can detect findings of a worsening obstruction. This may reduce the wait time for a more detailed imaging study (such as CT) before deciding between conservative and surgical management.
